# Supplementary material for: Optogenetic induction of subcellular Ca2+ events in megakaryocytes and platelets using a highly Ca2+-conductive channelrhodopsin
Source: Commun Biol. 2025 Oct 7;8:1433. doi: 10.1038/s42003-025-08924-w (PMC12504666; doi:10.1038/s42003-025-08924-w)

SUPPLEMENTAL INFORMATION

Optogenetic induction of subcellular Ca^{2+} events in megakaryocytes and platelets using a highly Ca^{2+}-conductive channelrhodopsin

Yujing Zhang[1], Jing Yu-Strzelczyk[2], Dmitri Sisario[1], Rebecca Holzapfel[1], Zoltan Nagy[1], Congfeng Xu[3], Chengxing Shen[3], Georg Nagel[2], Shiqiang Gao[2]*, Markus Bender[1]*

[1]Institute of Experimental Biomedicine–Chair I, University Hospital Würzburg, 97080 Würzburg, Germany

[2]Department of Neurophysiology, Institute of Physiology, University of Würzburg, 97070 Würzburg, Germany

[3]Department of Cardiology, Sixth People's Hospital, Shanghai Jiaotong University School of Medicine, Shanghai, China

*Authors for correspondence:

Markus Bender, e-mail: bender_m1@ukw.de

Shiqiang Gao, e-mail: gao.shiqiang@uni-wuerzburg.de

Supplemental Figures

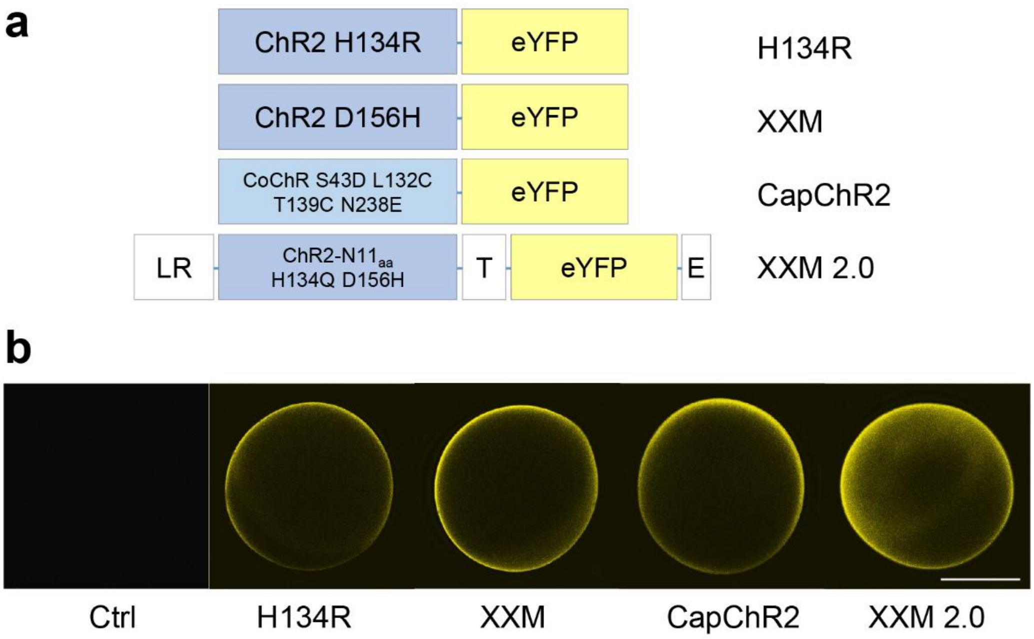

c

RFU	340 ± 17	484 ± 30	443 ± 23	664 ± 37

Supplementary figure 1. Expression of channelrhodopsin variants in Xenopus oocyte.

(a) Schematics of channelrhodopsin constructs used in this research. ChR2, Channelrhodopsin 2 from *Chlamydomonas reinhardtii*; eYFP, enhanced yellow fluorescent protein; CoChR, the channelrhodopsin from *Chloromonas oogama*; LR, the cleavable artificial signal peptide Lucy–Rho; -N11aa, truncation of the N terminal 11 amino acids; T, plasma membrane trafficking signal from Kir2.1; E, the endoplasmic reticulum (ER) export signal from Kir2.1. (b) Expression of different channelrhodopsins in Xenopus oocytes. Oocytes were injected with 20 ng cRNAs of each construct. After 3 days of expression, fluorescence imaging of oocytes was done using a confocal laser scanning microscope (LSM 5 Pascal, Carl Zeiss). Shown are single planes through the center of the oocyte. Scale bar = 500 µm. (c) The eYFP-tagged proteins were quantified by the relative fluorescence units (RFUs). For every sample, 6 oocytes were homogenized in 300 ul in Normal Frog Ringer (NFR) buffer and centrifuged at 30000 rcf to collect the pellet for fluorescence measurement. Fluorescence emission was measured at the range of 510–580 nm using the Quantus™ Fluorometer (Promega, Walldorf) with 495-nm excitation. The basal fluorescence value of control oocytes (125 ± 9) has been subtracted for the channelrhodopsin samples. n = 6, error bars = sd.

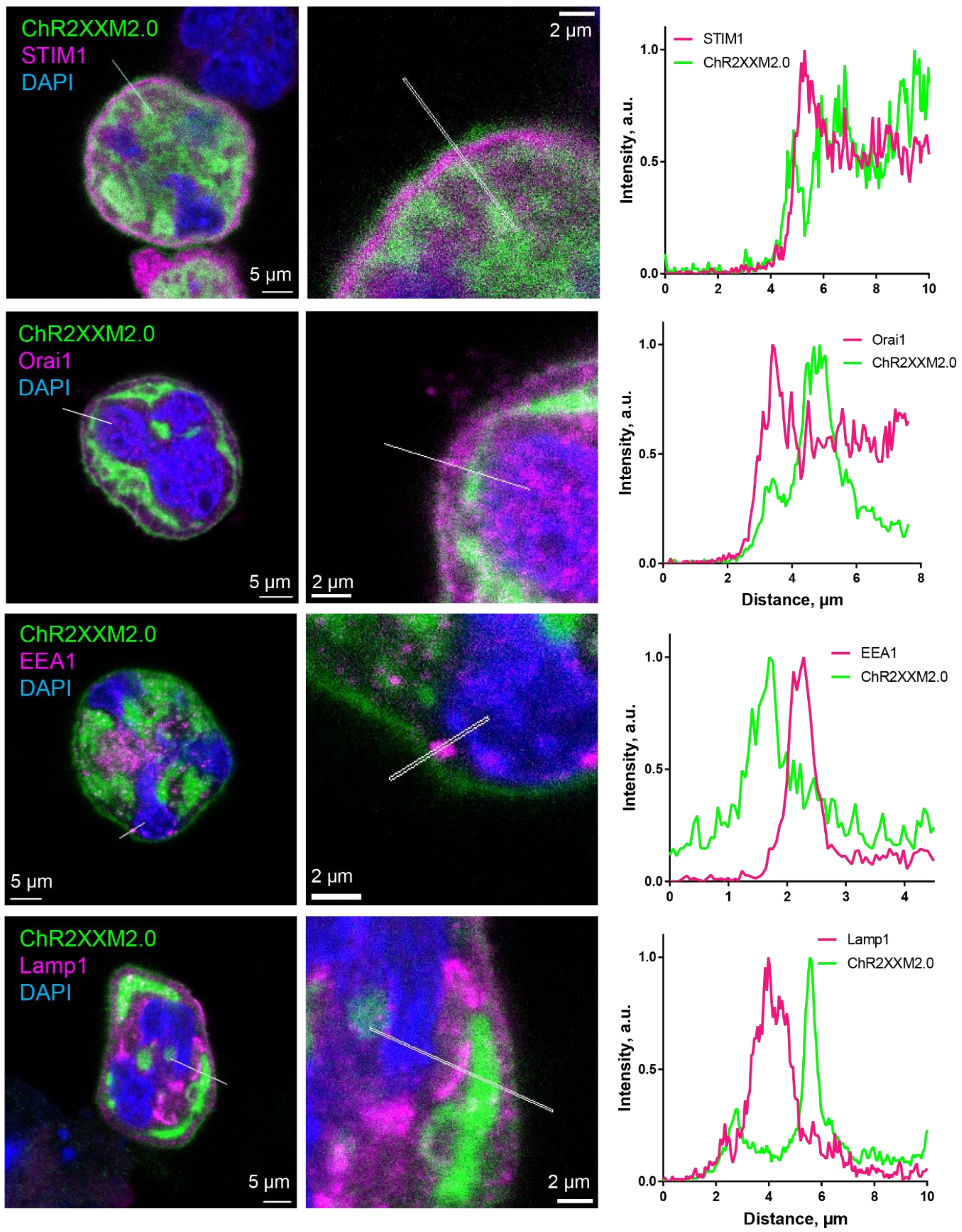

Supplementary figure 2: Assessment of subcellular localization of ChR2XXM2.0.

Representative ChR2XXM2.0 expressing megakaryocytes were fixed, immunolabeled with primary rabbit antibodies for STIM1 (11565-1-AP, ThermoFisher, 1:200), Orai1 (GTX16613, GeneTex, 1:200), EEA1 (C45B10, Cell Signaling, 1:400) or Lamp1 (ab24170, Abcam, 1:200) and stained with Alexa647-conjugated anti-rabbit antibodies (dilution 1:400) and DAPI. Co-localization (or lack thereof) of ChR2XXM2.0 and the indicated proteins is evident from the density profiles taken from the white line drawn through the respective images.

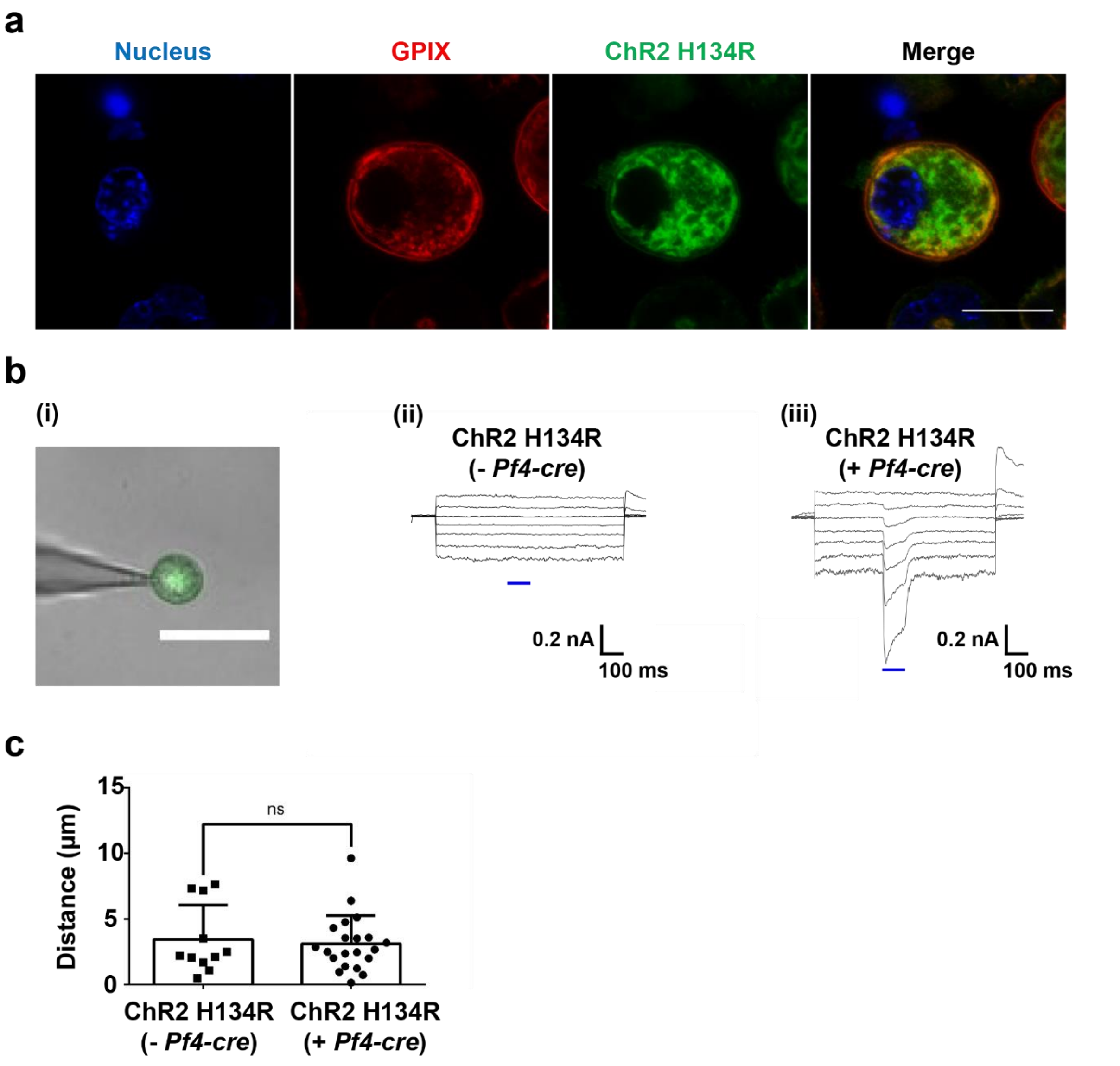

Supplementary Figure 3: Characterization of the ChR2 H134R variant.

(a) Representative confocal images of bone marrow-derived ChR2 H134R MKs, which were stained for the nucleus (blue) and GPIX (red). ChR2 H134R was visualized by its fusion protein EYFP (green). (b) (i) Image of whole-cell patch clamp of a MK from a ChR2 H134R transgenic mouse. Superimposed current traces for control (*Pf4-cre* negative) (ii) and ChR2 H134R (*Pf4-cre* positive) MKs (iii) after applying 100 ms light pulse (blue bar, 473 nm, 550 µW/mm^2) from a series of voltage pulses from −80 to +40 mV of 1 s duration with 20 mV increments from a holding potential of −40 mV. (c) Distance of polarized movement of control (*Pf4-cre* negative) and ChR2 H134R (*Pf4-cre* positive) MKs at 20 min after 3 min local illumination (n=11 to 21). Mann-Whitney U test. NS = no significant difference. Results are mean ± s.d.

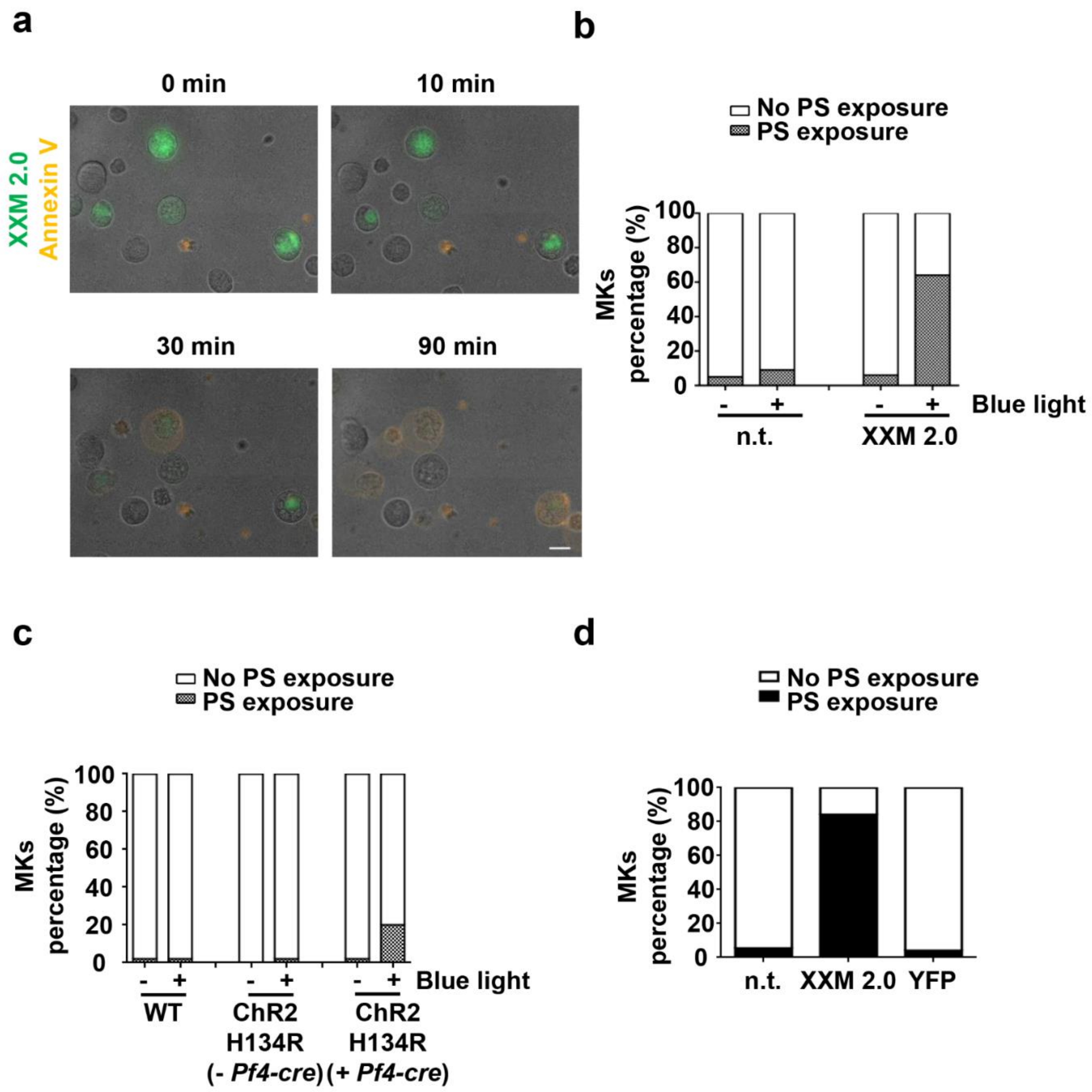

Supplementary Figure 4: Long-term global illumination induces PS exposure on ChR2 XXM2.0 MKs. (a) Time-lapse images of 90 min global illumination of ChR2 XXM2.0 MKs with LED light (488 nm, 100 µW/60 mm^2). Annexin V was used to stain phosphatidylserine. Scale bar indicates 22 µm. Determination of PS exposure percentage of (b) ChR2 XXM2.0 or non-transduced (n.t.) MKs or (c) ChR2 H134R (*Pf4-cre* positive), or control (*Pf4-cre* negative), or WT MKs during 90 min of LED-based blue light exposure. (d) Determination of MK PS exposure percentage of non-transduced (n.t.) MKs, ChR2 XXM2.0-expressing MKs and YFP-expressing MKs after 90 min illumination.

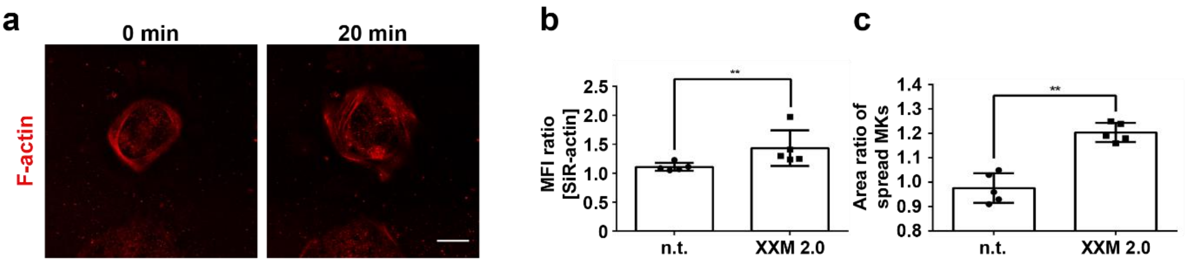

Supplementary Figure 5: Increased megakaryocyte spreading after global illumination.

(a) Spread MK was preincubated with 1 µM SiR-actin before illumination to visualize stress fiber formation. Observation was performed for 20 min after 3 min global illumination with blue (488 nm) light. (b) Quantification analysis of MFI ratio of SiR-actin (20 min post illumination / before illumination) (n=5). (c) The ratio of MK spreading area (20 min post illumination / before illumination) (n=5). Scale bar represents 22 µm. n.t. indicates no transduction; XXM 2.0: ChR2 XXM2.0-expressing MKs. Mann-Whitney U test. $^{**}P < 0.01$. Results are mean ± s.d.

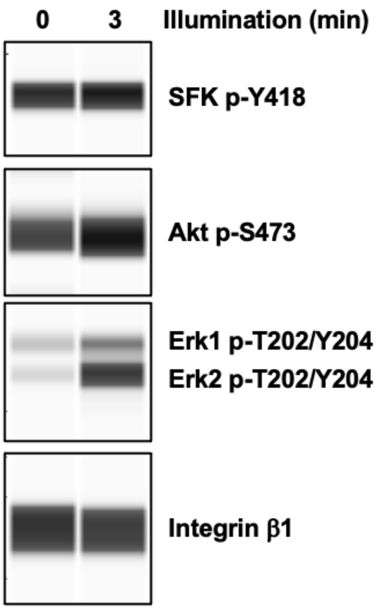

Supplementary Figure 6: Increased phosphorylation of SFK, Akt and Erk1/2 after global illumination.

Automated quantitative capillary-based immunoassay analysis of phosphorylation changes in Src family kinase (SFK, p-Y418, #6943), Akt (p-S473, #9271), and Erk1/2 (p-T202/Y204, #4370) in ChR2 XXM2.0-expressing MKs in suspension after 0 min and 3 min of global illumination. Integrin β1 (#34971) was used as a loading control. Protein lysates (1 mg/ml) were analyzed using the Jess instrument (ProteinSimple). Antibodies were purchased from Cell Signaling Technology and used at a 1:10 dilution.

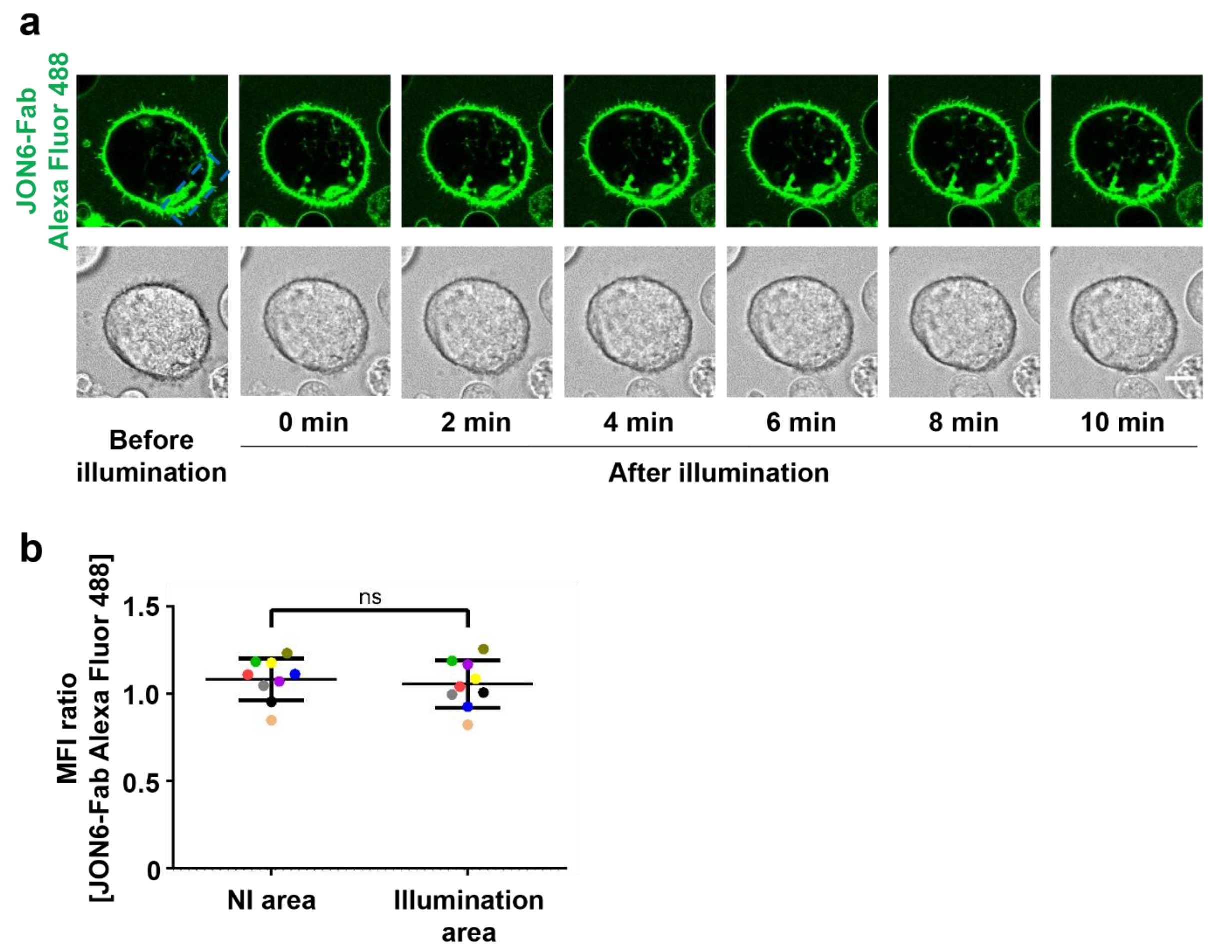

Supplementary Figure 7: Polarized light-induced Ca^{2+} influx does not affect integrin αIIbβ3 distribution.

(a) Spread ChR2 XXM2.0 expressing MK was incubated with 4 µg/ml JON6-Fab Alexa Fluor 488 before 3 min local illumination, followed by 10 min observation. The blue, dashed rectangle (upper left image) indicates illumination region. Scale bar indicates 10 µm. (b) Quantification of MFI ratio of bound JON6-Fab Alexa Fluor 488 at 10 min (post local illumination/before local illumination) on ChR2 XXM2.0 expressing MKs. MFI was determined of an illuminated area and a non-illuminated area of the same cell, which is indicated by same color code (n=9). Mann-Whitney *U* test. NS = no significant difference. Results are mean ± s.d.

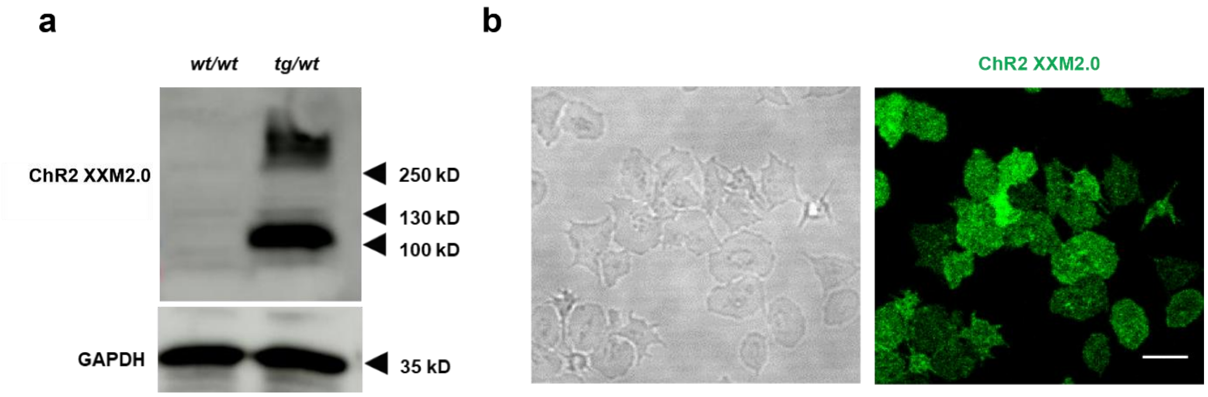

Supplementary Figure 8: ChR2 XXM2.0 is expressed in platelets of transgenic mice.

(a) Determination of expression of ChR2 XXM2.0 in platelet lysates. wt: wildtype, tg: transgenic. Bands probably indicate aggregation of the ChR2 protein. Anti-GFP (Cell Signaling, #2956S, 1:1000), anti-GAPDH (Cell Signaling, #2118S, 1:1000). (b) Confocal imaging of spread platelets on fibrinogen of homozygous (*tg/tg*) ChR2 XXM2.0 mice, the ChR2 XXM2.0 is visualized by the fluorescence of the fused EYFP tag. Scale bar indicates: 5 µm

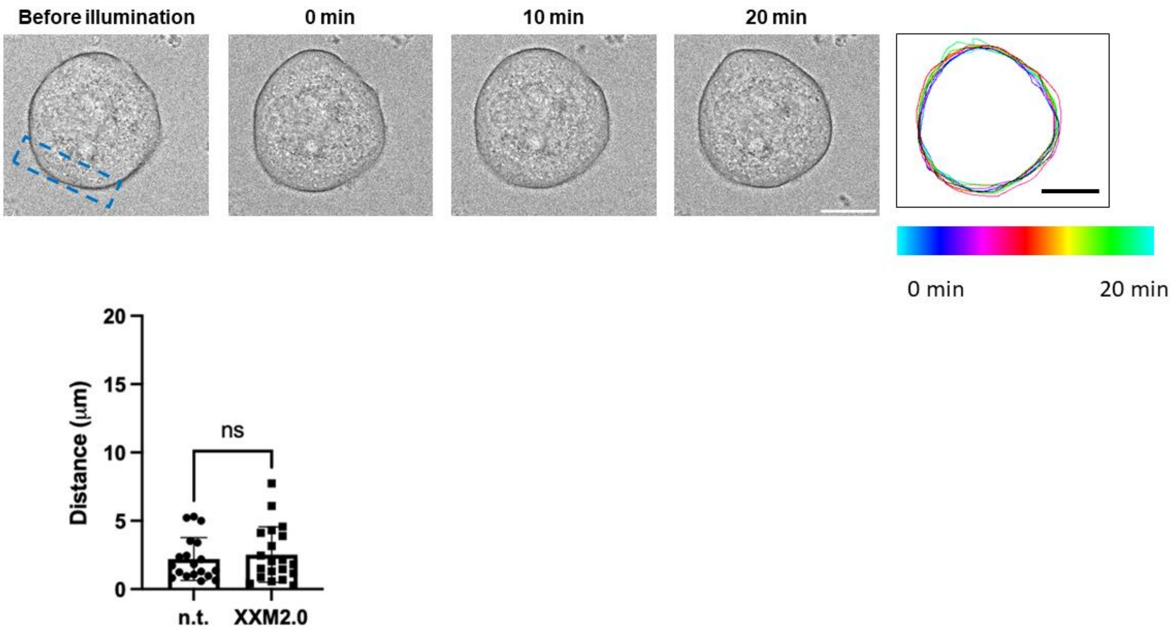

Supplementary Figure 9: Local illumination does not induce polarized movement of ChR2 XXM2.0 MKs on fibronectin.

Representative images of time-lapse microscopy of ChR2 XXM2.0 expressing MK on fibronectin, which was locally illuminated for 3 min and then observed for 20 min. Local illumination was performed with FRAP module of a confocal microscope. The blue, dashed

rectangle indicates illumination area. 0 min, 10 min, and 20 min mean the time point directly after 3 min local illumination. Color-coded (time) representative cell outlines are shown on the right. Scale bars: 22 µm. Distance of polarized movement of ChR2 XXM2.0 expressing MKs (XXM 2.0) and control MKs (n.t. indicates no transduction) post local illumination. The polarization distance was analyzed by the distance between the center of mass of MKs before 3 min local illumination and after 20 min observation. Results are mean ± s.d.

Supplementary Figures extended: uncropped Western blots

- **Figure 3a**

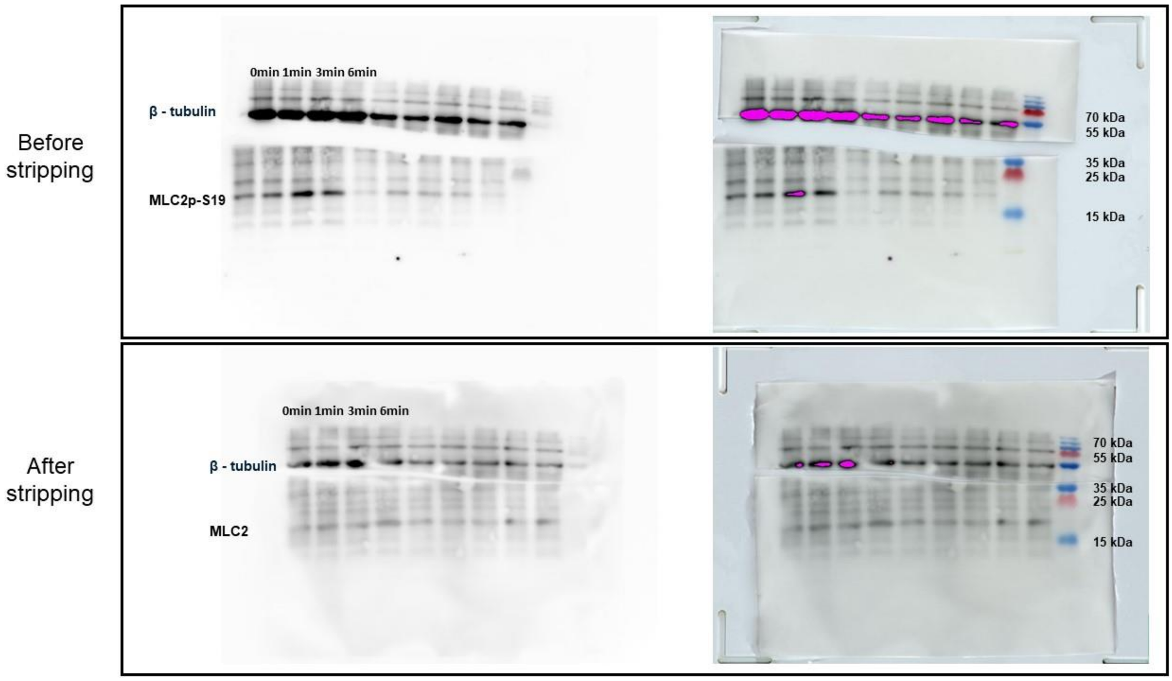

- **Supplementary Figure 8a**

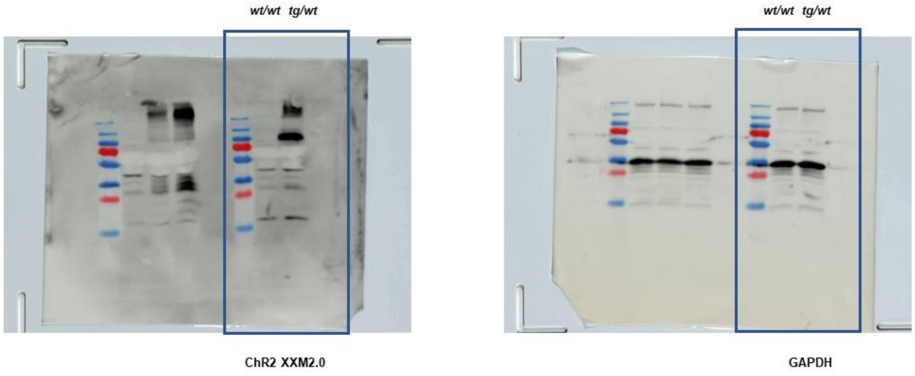

Supplement: Supplementary file 2 — Supplemental Information [file 42003_2025_8924_MOESM2_ESM.pdf]
