# Supplementary material for: Optogenetic induction of subcellular Ca2+ events in megakaryocytes and platelets using a highly Ca2+-conductive channelrhodopsin
Source: Commun Biol. 2025 Oct 7;8:1433. doi: 10.1038/s42003-025-08924-w (PMC12504666; doi:10.1038/s42003-025-08924-w)
Supplement: Supplementary file 3 — Description of Additional Supplementary Materials [file 42003_2025_8924_MOESM3_ESM.pdf]

Description of Additional Supplementary Files

File name: Supplementary Data 1

Description: Source data

File name: Supplementary Video 1

Description: ChR2 XXM2.0 expressing megakaryocytes spread on fibrinogen were preincubated with 10 µM Cal 590™ for 10 min before 3 min global illumination (488 nm) to visualize intracellular Ca2+ changes. Circle indicates illumination area

File name: Supplementary Video 2

Description: ChR2 XXM2.0 expressing megakaryocytes spread on fibrinogen were locally illuminated for 3 minutes and observed for 20 minutes. Blue rectangle indicates illumination area. First video without preincubation; second video with preincubation with 100 µM BAPTA-AM.

File name: Supplementary Video 3

Description: ChR2 XXM2.0-mkate2 expressing megakaryocyte on a fibrinogen coated surface was locally illuminated for 3 minutes and observed for 20 minutes. Blue rectangle indicates illumination area. First video: without fibrinogen in suspension; second video with 100 µg/mL fibrinogen in suspension to prevent polarized MK movement by blocking integrin αIIbβ3 binding to surface bound fibrinogen after local illumination.

File name: Supplementary Video 4

Description: ChR2 XXM2.0 expressing megakaryocytes spread on fibrinogen were locally illuminated for 1 minute and observed for 20 minutes. Blue rectangle indicates illumination area.

File name: Supplementary Video 5

Description: ChR2 XXM2.0 expressing platelets spread on fibrinogen were illuminated for 10 minutes. Blue rectangle indicates illumination area. Platelets were preincubated with 0.35 µg/ml Annexin V Alexa Fluor 546 to visualize phosphatidylserine and 18 µg/ml anti-P-selectin Alexa Fluor 647 antibody to visualize P-selectin.
